# A risk-mitigation approach to the management of induced seismicity

**DOI:** 10.1007/s10950-015-9478-z

**Published:** 2015-02-04

**Authors:** Julian J. Bommer, Helen Crowley, Rui Pinho

**Affiliations:** 1 Civil & Environmental Engineering, Imperial College London, London, SW7 2AZ UK; 2 Eucentre, Via Ferrata 1, Pavia, 27100 Italy; 3 Civil Engineering & Architecture, University of Pavia, Via Ferrata 1, Pavia, 27100 Italy

**Keywords:** Induced seismicity, Risk management, Seismic hazard, Fragility functions, Duration, Seismic retrofitting

## Abstract

Earthquakes may be induced by a wide range of anthropogenic activities such as mining, fluid injection and extraction, and hydraulic fracturing. In recent years, the increased occurrence of induced seismicity and the impact of some of these earthquakes on the built environment have heightened both public concern and regulatory scrutiny, motivating the need for a framework for the management of induced seismicity. Efforts to develop systems to enable control of seismicity have not yet resulted in solutions that can be applied with confidence in most cases. The more rational approach proposed herein is based on applying the same risk quantification and mitigation measures that are applied to the hazard from natural seismicity. This framework allows informed decision-making regarding the conduct of anthropogenic activities that may cause earthquakes. The consequent risk, if related to non-structural damage (when re-location is not an option), can be addressed by appropriate financial compensation. If the risk poses a threat to life and limb, then it may be reduced through the application of strengthening measures in the built environment—the cost of which can be balanced against the economic benefits of the activity in question—rather than attempting to ensure that some threshold on earthquake magnitude or ground-shaking amplitude is not exceeded. However, because of the specific characteristics of induced earthquakes—which may occur in regions with little or no natural seismicity—the procedures used in standard earthquake engineering need adaptation and modification for application to induced seismicity.

## Introduction

The phenomena of induced and triggered seismicity, which include all earthquakes whose time and location are related to some anthropogenic activity, have been recognised for many decades. Well-known examples have included earthquakes associated with the impounding of deep reservoirs (e.g., Simpson et al. 1988) and with mining, among others (e.g., Klose 2013). The topic of induced seismicity has attracted greater attention in recent years, particularly because of several cases of seismicity related to processes involving the high-pressure injection of fluids into the Earth’s crust, including waste-water disposal (Ellsworth 2013), enhanced geothermal systems (Majer et al. 2007) and hydraulic fracturing for shale gas production (Davies et al. 2013). Recognition of the link between such processes and induced earthquakes is clearly not new, the relationship having been well proven in the case of waste-water disposal at the Rocky Mountains arsenal in the late 1960s (Healy et al. 1968). The heightened focus on induced seismicity in recent years has been due to a number of factors, including greater frequency of cases (and wider reporting of these in the media) and the fact that some of the induced earthquakes have occurred in densely populated areas, such as the December 2006 earthquake caused by the Basel Deep Heat Mining project (Deichmann and Giardini 2009). An additional factor may be the broader controversy associated with some of these processes—particularly hydraulic fracturing (or ‘fracking’)—despite the fact that fracking has been observed to be the cause of very few felt earthquakes, and the few that have occurred have been of small magnitude (Davies et al. 2013; NRC 2012). Moreover, there has been a degree of ‘cross-contamination’, whereby concerns regarding one particular source of induced seismicity has led to increased public, regulatory and media attention on all anthropogenic causes of earthquakes, with the concomitant blurring of the specific technical issues in each case. The seismogenic potential of each technology and also each geological setting should be assessed individually, since the conditions for producing earthquakes may vary considerably from one anthropogenic activity to another, as well as from one location to another.

There is clearly a need to address and effectively manage seismicity that might be induced by a wide range of human activities, several of which are related to meeting humanity’s ever-growing demand for energy. A framework for the management of induced seismicity would be of direct benefit to operators wishing to fulfil their social and environmental responsibilities while avoiding interruption of their activities, and to regulatory bodies charged with protecting the public against potentially adverse effects of such activities. Until now, the key focus has been on controlling or limiting the induced earthquakes, usually in terms of the largest magnitude of any induced or triggered earthquake. Since the occurrence of induced earthquakes is directly related to human activities, the attraction of this approach—which is not available when confronting the threat of natural (tectonic) earthquakes—is obvious, since control of the causative activity could be expected to result in control of consequent seismicity. To date, the success of such control systems on induced seismicity in practice has been very limited, even though some impressive schemes have been developed and calibrated retrospectively (see “Section 2.1”). Our view is that approaches based on control of the induced seismicity are far from reaching a state of development whereby they could be relied on with great confidence.

In this paper, we propose an alternative paradigm for the management of induced seismicity, which moves away from the concept of controlling the number, frequency or magnitude of the induced earthquakes and focuses instead on the consequences of the earthquakes that may occur. In effect, the approach is similar to that which is routinely adopted for managing natural seismicity: accepting that the earthquakes may occur, quantifying their effects, and taking appropriate measures to mitigate the negative consequences of these effects on the built environment. In many regards, this approach takes advantage of the tools developed and applied to natural seismicity over many decades, but it is also noted that most of these require modification for application to induced earthquakes. This need for adaptation arises both due to the specific characteristics of induced earthquakes and because induced earthquakes may occur in regions where there is very little natural seismicity, and hence, the built environment may be particularly susceptible to ground shaking. In the development of risk models for induced seismicity, there are currently several important knowledge gaps. For many of these, the approaches used in conventional earthquake engineering (for ground-motion prediction models and fragility functions, for example) may be adapted provided that there is access to local data. The greatest knowledge gap is probably related to the development of the hazard models and, specifically, the models for occurrence of future earthquakes in these non-stationary processes. However, we devote relatively little space in this paper to this issue, partly because we believe that bespoke solutions will need to be developed for each setting and technology, but also because it is our view that it is precisely because of the large uncertainty associated with predictive models for induced seismicity that a risk management approach is most appropriate. The paper begins with an overview of the key elements of risk and the options for reducing risk by modification of each element. This is followed in “Section 3” by a discussion of the challenges related to quantifying and managing non-physical risk in terms of disturbance due to ground shaking. This is followed in sections 4 and 5 by more detailed discussions of the quantification of induced seismic risk and engineering measures to mitigate that risk. We conclude with a brief summary and some suggestions for decision-making frameworks to address different levels of induced seismic risk.

## Options for mitigating seismic risk

Seismic risk can be defined as the likelihood or probability of different levels of undesirable consequences due to the occurrence of earthquakes. Such consequences may include loss of life, injury, damage and collapse of buildings, economic costs, and business interruption, among others. For the specific case of induced seismicity, the consequences could also include annoyance of the affected population, non-structural damage to buildings and reputational damage to the operator of the activity responsible for the earthquakes. The ultimate objective of any effective program for the management of induced seismicity must be to limit the consequent seismic risk.

In simple terms, seismic risk can be considered as the convolution of four factors:

1 $$ \mathrm{SEISMIC}\ \mathrm{RISK}=\mathrm{SEISMIC}\ \mathrm{HAZARD}*\mathrm{EXPOSURE}*\mathrm{FRAGILITY}*\mathrm{CONSEQUENCE} $$

In Eq. 1, the seismic hazard is the quantification of the earthquakes, for which the magnitude of the events alone is not sufficient. Generally, the hazard will be defined by a measure of the ground shaking, and in order to quantify the likelihood of the risk, the associated frequency or probability of exceedance. The exposure refers to the characterisation of the built environment—including dwellings, commercial and industrial buildings, and all infrastructure elements (utilities and transportation)—and the inhabitants in the area where the perceptible shaking may occur. The fragility defines the susceptibility of each element of the exposure to be damaged and cause undesirable consequences under different levels of ground shaking. The combination of the fragility and consequence functions defines vulnerability. The model for the consequences will reflect the metric chosen to represent the risk, such as the number of people adversely affected or the economic impact.

**Fig. 1:**
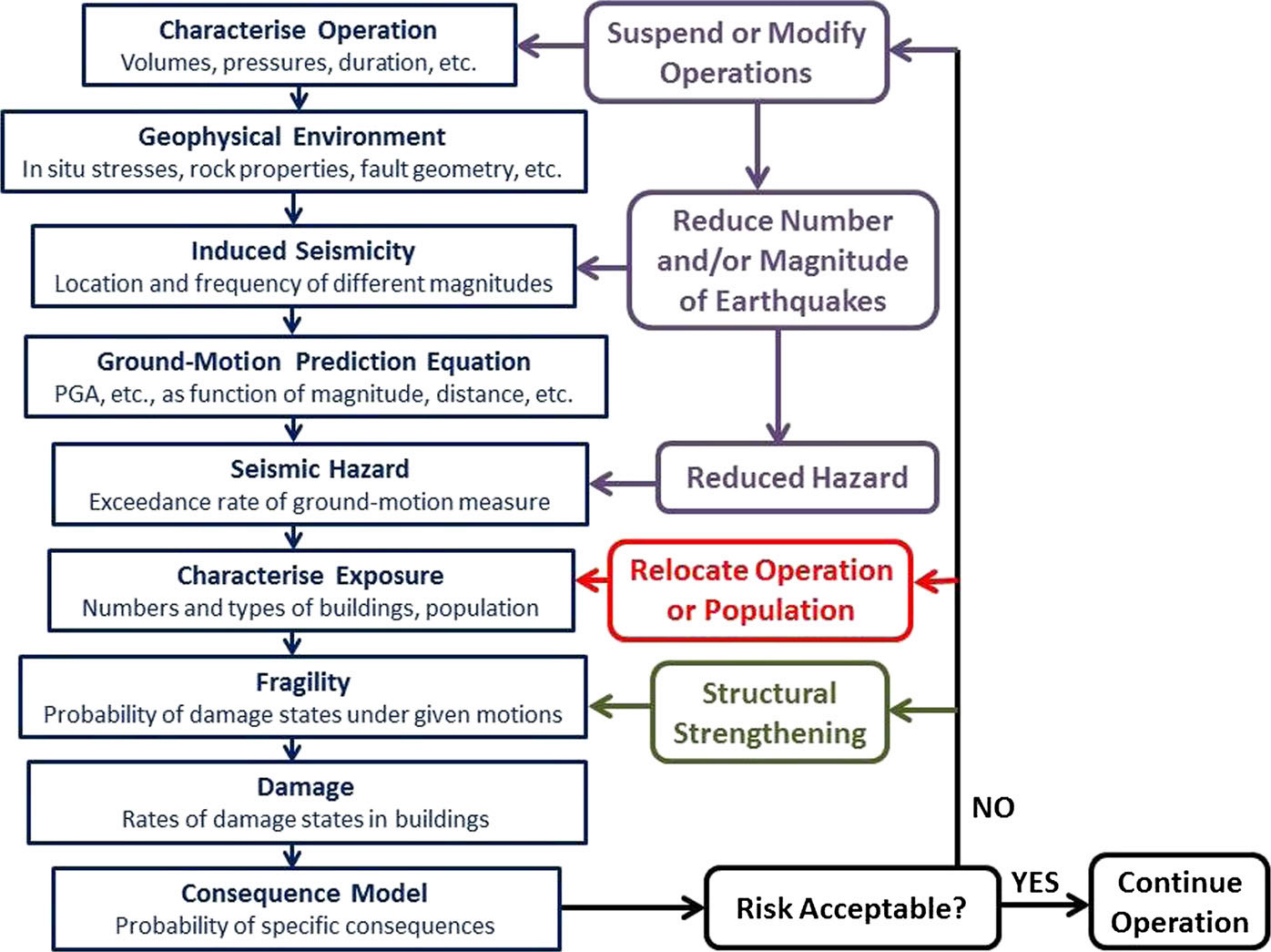
The sequence of steps involved in estimation of induced seismic risk and three options (*purple*: control hazard; *red*: modify exposure; *green*: modify fragility) for mitigation if the risk is found to exceed tolerable levels

One key advantage of a risk-based approach to managing induced seismicity becomes immediately apparent, since an operation conducted in a remote and uninhabited region (i.e., zero exposure) need not be overly concerned by induced earthquakes, other than the impact they might have on the facilities associated with the operation itself. By contrast, the occurrence of even small-magnitude induced earthquakes in a populated region without any appreciable natural seismicity—which is likely to result in a building stock that is susceptible to lateral loading and a population that is sensitive to being shaken—may result in elevated risk despite the relatively low level of seismic hazard. The steps involved in estimating the risk are illustrated in Fig. 1. In order to limit or reduce the risk, one or more of the first three factors in the right-hand side of Eq. 1 need to be limited or reduced in proportion, as illustrated in the same figure. The options are not mutually exclusive, and a management strategy might involve efforts to control two or even all three elements. In the following sub-sections, the options, advantages and challenges associated with control of each of the three elements of induced seismic risk are discussed individually.

### Control of earthquake hazard

In this discussion, we make the implicit assumption that the issue of the originating cause of any induced seismicity is not under discussion and that the operator would assume full responsibility; for an excellent discussion of discriminating between induced and natural seismicity, we refer the reader to Dahm et al. (2013). However, a very brief discussion is warranted here of the distinction between induced and triggered seismicity, for which we adopt the definition that triggered earthquakes are those where the stress change leading to the event is only a small fraction of the ambient level (in other words, the earthquake was incipient and its time of occurrence brought forward by the anthropogenic activity) whereas, for induced seismicity, the stress change is comparable in magnitude to the ambient shear stress acting on a fault (McGarr et al. 2002). In operations that involve high-pressure injection of fluids, small-magnitude earthquakes caused directly by hydraulic fracturing would be considered induced, whereas larger events caused by the injected fluid intersecting a critically stressed pre-existing fault would be triggered. In this regard, it would be expected that a model would be developed for the hazard from the induced seismicity, whereas the intention might be to eliminate the hazard of triggered seismicity through identification and subsequent avoidance of significant faults.

In regions where moderate-to-large magnitude earthquakes may occur within the natural patterns of seismicity, the risk-based approach proposed herein may raise questions regarding the importance of identifying individual events as having been triggered by human activities. If these are earthquakes that would have occurred at some (unknown) point in time because of the regional tectonics, then the issue of why adequate seismic protection was not already in place is surely more important than discussions related to the small probability that the exact timing of the events was influenced by hydrocarbon production or another anthropogenic activity.

As noted earlier, in the case of induced seismicity, given that the earthquakes are being caused by human activities, an option may exist that is not available when confronting natural seismicity, namely, to control the occurrence of the earthquakes and thereby limit the seismic hazard. The approach that has generally been adopted in this regard is to implement ‘traffic light’ systems, which require real-time monitoring of the seismicity and pre-defined thresholds for acceptable levels of motion. The traffic light system would normally define a ‘green’ level, indicating that the induced earthquakes, if any, are not causing a concern and operations may continue unhindered; ‘amber’ to indicate that the levels are escalating towards unacceptable levels and the operations need to be modified; and ‘red’ to indicate that immediate suspension of the operations is required. There have been proposals for traffic lights in which the thresholds are defined only in terms of the magnitude of induced earthquakes (Green et al. 2012), although the relationship to damage potential is much better defined in terms of ground-motion characteristics. For an enhanced geothermal project in El Salvador, Bommer et al. (2006) developed a traffic light system defined by threshold of peak ground velocity (PGV), although these were translated into equivalent magnitudes since all events were expected to occur at comparable depths. This traffic light system was adapted for the Basel Deep Heat Mining project (Häring et al. 2008), and such approaches have been recommended for all enhanced geothermal projects (Majer et al. 2012) and for waste water injections as well (Zoback 2012).

To date, the implementation of traffic light systems has not been particularly successful, not least because in enhanced geothermal systems the largest earthquakes have tended to occur after shut-in of the pumping operations (Majer et al. 2007). Important work has been undertaken to model the post shut-in response of geothermal reservoirs (e.g., Baisch et al. 2006; Barth et al. 2013), and more advanced traffic light systems have been developed that can accommodate short-term changes in the seismicity (Bachmann et al. 2011; Mena et al. 2013). Mignan et al. (2014) have proposed the implementation of such an advanced traffic light within a risk-based framework, proposing in effect that the thresholds be related to potential losses but still focusing on risk mitigation through control of the hazard. Douglas and Aochi (2014) propose a scheme for controlling hydraulic stimulations in enhanced geothermal systems based on estimated risk (of disturbance to the local population through felt shaking); again, even though a risk framework is proposed, the focus is still exclusively on control of the hazard.

The traffic light concept is attractive for several reasons, not least because it provides a low-cost solution for risk mitigation, although that needs to be balanced against the economic implications of diminishing or suspending the operation causing the seismicity. The fact is that, until now, there has not been an application—in the forward sense rather than a retrospective analysis—of a traffic light system that has been successful in limiting the impact of induced earthquakes. For such a system to be effective, it would seem that very detailed knowledge of the in situ state of the crust where the injections are to be made is required, and also that the system has a rapid response to changes in pumping rates or volumes. Another issue that needs to be recognised is that traffic lights have only been proposed so far for operations involving the high-pressure injection of liquids into the Earth’s crust, and it is questionable whether the approach would even be feasible for other causes of induced seismicity, such as fluid extraction (particularly of gases), where there may be spatial and temporal delays as pressures change and stablise.

Until traffic light systems are implemented and proven in practice to provide a high degree of confidence that the hazard can be effectively controlled, to depend on such measures alone is a risky option. Even with high confidence in the ability of the system to avoid the occurrence of potentially damaging earthquakes, unless other measures for physical mitigation of the risk are taken, the thresholds will be dictated by the current state of the exposed built environment. If the local building stock is vulnerable to seismic damage, then the resulting thresholds may lead to unacceptably frequent interruption of productive activities, and this could render the whole operation economically untenable. If the operation potentially causing earthquakes is of sufficient economic value, there may be justification for mitigating the risk more reliably, through control of exposure or vulnerability, as discussed in the following sections.

A final note regarding limitations of hazard control approaches to induced seismicity is that, for regulated industries (such as hydrocarbon production), volumes and rates of fluid extraction may be imposed by license conditions; such a situation effectively renders a traffic light system redundant. We do not in any sense wish to discourage the valuable ongoing work to develop and improve such systems, but we believe that they cannot be a panacea for all cases and causes of induced seismicity, and even where applicable they should not be relied on as the sole tool to mitigate the attendant risk.

### Modification of exposure

From Eq.1, it can be immediately appreciated that risk only exists if there is spatial coincidence of the hazard (ground-shaking) and exposure (buildings, infrastructure and population). Induced seismicity tends to occur in rather close proximity to the causative operations, and since earthquake ground-shaking attenuates rapidly with distance from the source, increased separation of the operations from population centres is a reliable way to reduce the associated seismic risk. Clearly, the easier way to achieve this is to select a remote location for the operation, which may be a possibility for activities such as waste water disposal, for example. However, for many activities, particularly those related to hydrocarbon production or energy production, the options for relocation are severely limited. Moreover, there may be compelling reasons to locate operations within a heavily populated area, a case in point being the Basel Deep Heat Mining project, which in addition to energy generation needed to provide district heating—which cannot be provided efficiently over long distances—in order to be economically viable.

If the relocation of the earthquake-inducing operation is not an option, the only way to separate the hazard and exposure is through relocation of the exposed population. This is unlikely to be a viable option in most cases, particularly if the affected area is extensive, as would be the case for a major hydrocarbon field for example. If, however, there is a very small exposed population situated in the area of highest hazard, relocation—with sufficient incentives, such as economic benefits and improved living standards at the new address—may be an expedient option. Although wholesale relocation of affected populations will generally not be a viable solution for risk management, the option of relocating a small proportion of the population living in extremely vulnerable dwellings—and demolishing those houses—should certainly be considered among the options for limiting the seismic risk.

### Vulnerability reduction

Structural strengthening to reduce seismic vulnerability is the approach used to mitigate seismic risk due to tectonic earthquakes, since the associated hazard cannot be controlled and exposure is generally driven by other considerations. Such an approach is also attractive for the mitigation of induced seismic risk because the technology for enhancing earthquake resistance of structures is well established.

Moreover, the application of such measures brings an assured reduction of risk with considerably less uncertainty than any programme of hazard control. However, the costs of structural upgrading in large numbers of buildings can be considerable, even if the interventions are relatively modest and designed only to prevent collapse. Therefore, this option will be feasible where the economic benefits of the operation that could potentially generate earthquakes are sufficient to justify the costs. The costs of structural strengthening also need to be balanced against the risks of not taking such measures, including the loss of all investment in the operation if induced earthquakes lead to abandonment of the project due to public pressure or regulatory intervention.

Another consideration is that, any extensive upgrading work will be disruptive to the occupants of a building, and this inconvenience needs to be considered in the design and implementation of a strengthening programme. Experience has shown that occupants will be generally much more receptive if the strengthening measures are accompanied by some enhancements of the living or work space provided by the buildings.

Our view is that, in many cases, structural strengthening will be an indispensable element of managing induced seismicity. A point to be emphasised at this stage is that in many cases the level of shaking from the small-magnitude earthquakes induced by anthropogenic processes will only lead to non-structural damage, such as plaster cracks. Structural interventions to prevent such minor damage would be extremely difficult and could not possibly be cost effective; if the physical separation between the seismicity and the exposure cannot be increased, then, in such cases, compensation for the repair of the damage may be the only viable recourse. It should also be acknowledged that there will often be an element of non-physical risk as a consequence of induced earthquakes—which may also pose a serious threat to causative operations—and so for completeness this issue is first discussed briefly in the next section.

## Quantification and mitigation of non-physical risk

Induced seismicity is often limited to small-magnitude earthquakes, which pose a minor threat in terms of physical damage but may be clearly felt by the exposed population. The anxiety, fear and annoyance that may result from such shaking episodes—particularly if these are frequently repeated and appear to be escalating (giving rise to fears of larger earthquakes)—can be expected to lead to a negative response from the affected population, especially since the shaking will be viewed as an imposed risk. The sensitivity of any given population to such shaking episodes will depend on many specific aspects of local conditions, including the levels of natural seismicity to which the local population is accustomed. Quantifying the tolerance to induced shaking is likely to prove challenging, especially since there is very little guidance on this topic. For the Berlín enhanced geothermal project, Bommer et al. (2006) inferred tolerable thresholds from guidance on other sources of man-made vibrations together with consideration of the vulnerability of the local building stock, but it would be inadvisable to rely too heavily on such thresholds as a basis for defining tolerance levels for shaking from induced earthquakes. In developing their protocol for stimulation control in geothermal projects, based on limiting the risk of exceeding tolerable levels of felt motions, Douglas and Aochi (2014) acknowledge the difficulties in defining reliable thresholds of intensity and frequency of shaking caused by induced earthquakes.

More important than quantifying the tolerance levels and the non-physical risk are appropriate measures to mitigate this risk. Experience from several projects has demonstrated that people will be more tolerant of shaking of which they have been forewarned, and this tolerance will be further increased if the benefits of the operation are clearly conveyed. Therefore, transparent and open communication with the affected population should be viewed as indispensable elements of any programme to manage induced seismicity. This communication should also include the estimates of hazard and risk, although such information can be difficult to convey to the public when it is framed in probabilistic terms and associated with very large uncertainties. However, if structural strengthening measures are part of the risk management programme, then this can be very positively communicated to the local population and would reduce the impact of the uncertainty in the hazard estimates and any reliance on a traffic light system. Equally, if the physical risk can confidently be demonstrated to be low, without structural intervention, then there can be clear benefit in communicating such a conclusion provided this can be done in a clear and accessible manner.

## Assessment of physical risk

As stated above, our view is that the most appropriate response to the potential of induced seismicity is to assess the physical risk posed by potential ground shaking, and then to apply structural strengthening measures to reduce the risk to acceptable levels. In this section, we outline the various steps required for such a quantification of the risk. As has been noted previously, procedures for most of these elements are well established in the field of earthquake engineering to mitigate the risk from natural earthquakes, but adaptations are required for their application to the special case of induced seismicity.

The scope is limited to ground shaking effects on buildings, but the same principles would apply to collateral hazards, such as liquefaction, and other exposed elements, such as bridges, etc.

### Establishing the baseline risk

Ideally, before operations commence that could potentially lead to induced seismicity, the baseline hazard and risk from natural (tectonic) seismicity should be established. Such a baseline allows for more informed decision making regarding the potential increase in risk as a result of the anthropogenic activity. Clearly, it is also desirable to estimate the seismic hazard that might result from induced earthquakes, although the uncertainty will inevitably be very large before any real-time monitoring has begun. An important feature of induced seismicity, however, is that new data are likely to be accumulated far more rapidly than might be the case for natural earthquakes, allowing more frequent updates of the hazard estimates (“Section 4.8”). The component of the risk that can be estimated with greater confidence *a priori* is the characterisation of the exposed building stock and its associated seismic vulnerability. There is a very obvious advantage in assessing the state of the exposed building stock before any induced earthquakes occur, in order to identify any pre-existing weaknesses or damage that warrant immediate attention and which may otherwise be erroneously attributed to the new shaking episodes.

### Selection of risk metrics

As discussed in “Section 2”, risk is commonly defined as the probability of loss. However, there are many quantitative measures of seismic risk (i.e., risk metrics), as loss may refer to a number of harmful human, social, economic and environmental consequences caused by damage; examples include loss of life, injury, repair costs, business interruption and loss of livelihood. The quantification of loss from a risk assessment may refer to the probabilistic distribution of loss conditioned on a given seismic event (from a so-called deterministic or scenario-based approach), or to the probability that the loss will equal or exceed specified values (at a site, at several sites or in an area) during a specified exposure time (see “Section 4.6” for a discussion on these two risk assessment methods). The latter loss metric is preferable for the purposes of risk mitigation as it considers all potential events that could impact the exposed population, along with their associated probabilities of occurrence. On the other hand, the scenario-based approach only considers one or a few events (usually chosen arbitrarily), which might each have a very low annual probability of occurrence and thus intervention to deal with these estimated losses might not be justified from a cost–benefit standpoint. Nevertheless, scenario-based risk assessment can be of use for emergency response planning, or for raising awareness of the importance of seismic risk mitigation.

From the outset of a risk assessment, it is important for all interested and affected parties to deliberate on the metrics that will be necessary input for decision-making, and that should be addressed in the analysis. In addition to the organisation undertaking the risk assessment, interested and affected parties might also include legislators, regulators, industry groups, environmentalists and citizens’ groups, amongst others: “*A risk characterisation that fails to address their questions is likely to be criticised as irrelevant or incompetent*, *regardless of how carefully it addresses the questions it selects for attention*” (Stern and Fineberg 1996). From a scientific viewpoint, the selected metrics of the risk assessment will condition the areas of the hazard, exposure, fragility and consequences that should receive additional attention, and for which the epistemic uncertainties should be reduced. For example, a focus on loss of life would require the exposure model to adequately model the number of people in and around buildings at different times of the day; a focus on economic loss will require the fragility functions to cover a range of damage states that would each require different repair techniques.

**Fig. 2:**
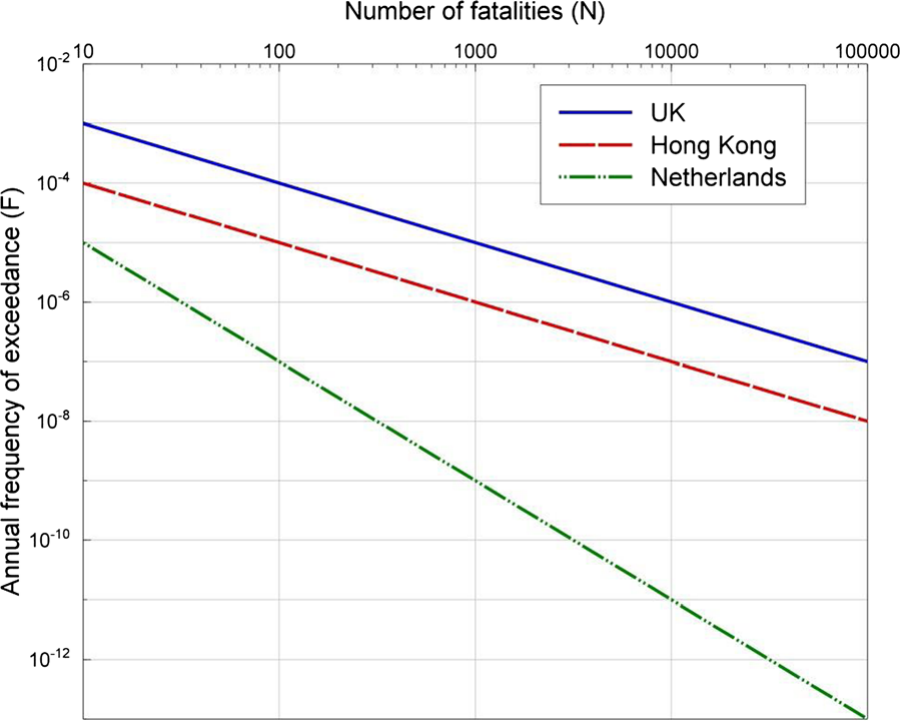
FN-curves proposed by various international standards (reproduced from Jonkman et al. 2003)
- Loss exceedance curves (or FD-curves, where D refers to economic damage) are frequently used when economic losses (such as repair costs and business interruption) are assessed and, similarly to the FN-curve, provide the annual frequency (or probability) of exceedance as a function of the economic loss (in monetary terms) (see Fig. 3).

As mentioned above, risk metrics can cover a number of measures of loss. Jonkman et al. (2003) provide an overview of 25 quantitative risk measures for loss of life and economic damage. A summary of the most common risk metrics, which as mentioned previously should not (and in some cases cannot) be based on single deterministic scenarios, is provided below:

- Individual risk is defined as the probability that an average unprotected person, permanently present at a certain location, is killed due to an accident resulting from a hazardous activity (Jonkman et al. 2003). This is more correctly referred to as the location risk since the risk to an individual will depend on the different locations where they spend time.
- Group risk has been defined as the relationship between frequency and the number of people suffering from a specified level of harm in a given population from the realization of specified hazards (IChemE 1985). Group risk is often represented through an FN-curve, which gives the annual frequency of exceedance as a function of the number of fatalities, on a log–log scale. These curves can then be compared with international standards, which define thresholds to the FN-curves (see Fig. 2).

**Fig. 3:**
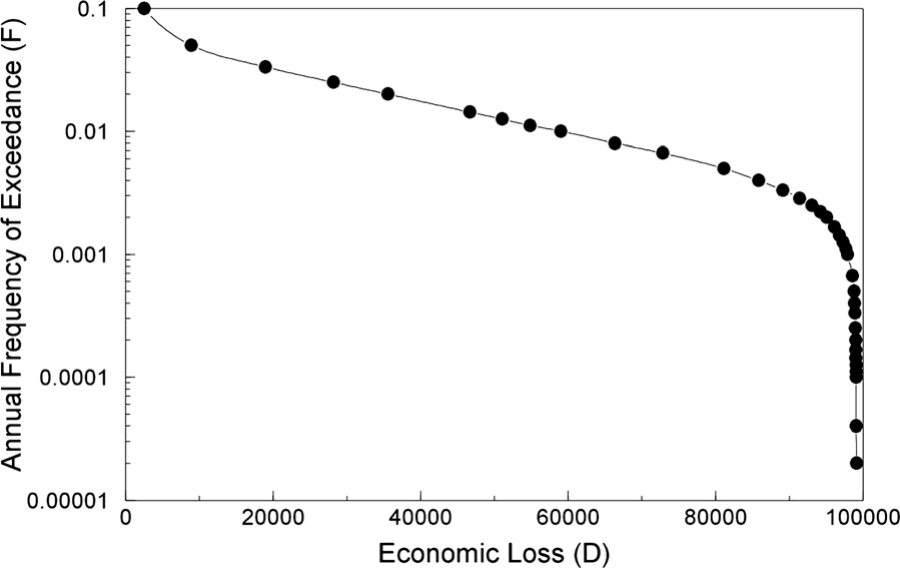
Example loss exceedance curve
- Average annual loss (also known as expectation value or expected value) provides the average number of losses per year and is given by the area under the FN- or FD-curve, for fatalities and economic losses, respectively.

Location risk and FN- and FD-curves allow the risk from induced seismicity to be compared with the risk due to other natural and man-made hazards. Thresholds of locations risk have been established in many countries (and can depend on whether the exposed element is new or existing and whether individuals have any control over their exposure to the hazard, or not), and as presented previously in Fig. 2, a number of international standard F-N curves define zones of unacceptable or intolerable risk (i.e., the area above the FN-curves shown in Fig. 2). Many countries also define zones where the risk is deemed to be so low as to be acceptable/negligible and zones where the risk should be reduced to be as low as reasonably practicable/achievable (ALARP/ALARA), ideally through a cost–benefit analysis. The equation for the threshold FN-curve for unacceptable/intolerable risk is generally given by *C*/*x* ^*n*^, where *x* is the number of fatalities, *C* is an anchor point and *n* gives the slope of the curve; if *n* is equal to 1, the standard is defined as risk neutral, whereas slopes greater than 1 are termed risk averse (Vrijling and van Gelder 1997).

The harmful consequences summarised above can all be linked to damage to the built environment. Hence, regardless of the risk metric chosen, it is always necessary to begin a risk assessment with an analysis of the physical damage caused by induced seismicity. Dividing the risk assessment into these two model components, the first estimating the damage to the built environment (through fragility functions, as discussed further in Section 4.5) and the second correlating the damage to consequences (which can be human, social, economic or environmental), ensures that the latter model, which suffers from higher levels of uncertainty, does not obscure the underlying drivers of the risk.

As has been noted previously, when only low levels of ground shaking are expected, and the exposed building stock does not include structures with extremely poor lateral resistance, the consequences will be limited to minor, non-structural damage. While not posing an important threat to occupants, such damage will nonetheless cause irritation and even distress to those affected, and repairs will be required. In such a case, the risk needs to be quantified in terms of costs of the repairs, which are likely to be paid by the operators or insurers. The appropriate tool in such a case is F-D curves, but calibration of the costs can be challenging. In an assessment of the risk generated by the operation of a geothermal field in Basel, Switzerland, the economic (repair cost) losses due to future potential events were estimated using the insurance payments from a previous event that occurred in December 2006 (Baisch et al. 2009). However, the large insurance claims settlements that followed the 2006 event did not necessarily reflect extensive damage, and the final ‘cost’ of the event is likely to have been influenced by contributions from pre-existing non-structural damage plus at least some cases of what the insurance industry calls ‘moral hazard’.

### Characterising induced seismic hazard

As has already been noted, characterising induced seismic hazard only in terms of the size (magnitude) of the possible earthquakes is of limited value. The impact of an earthquake on the built environment depends not only on its magnitude but also on the style-of-faulting, the focal depth, the distance of the source from any exposed buildings and the site conditions at the location of those buildings, all of which collectively determine the nature of the ground shaking. An even less helpful focus for induced seismicity, in which a series of earthquakes may be caused, are estimates of the largest earthquake that might occur (*M*_max_), albeit that this is likely to be an issue of intense public interest. In addition to the fact that such estimates are likely to be highly uncertain, the focus on these low-probability events ignores the risk posed by smaller but more frequent earthquakes that generally contribute significantly more to the risk.

The hazard should therefore be characterised in terms of parameters that characterise the ground shaking. Mignan et al. (2014) adopted macroseismic intensity for the assessment of induced seismic risk, arguing that this parameter facilitates communication of the hazard and risk. While this may be true, there are several shortcomings associated with the use of intensity in hazard and risk calculations, including the fact that the normal (as opposed to lognormal) distribution of residuals in predictive equations for intensity leads to hazard estimates that are not consistent with those obtained with ground-motion prediction equations (GMPEs) for instrumental parameters. Additionally, it is only possible in this case to derive empirical fragility functions in terms of intensity, and this is not our recommended approach for developing a risk model (see “Section 4.6”). Moreover, the quantitative reduction of risk through different structural strengthening measures could not be meaningfully modelled in terms of macroseismic intensity.

The choice of ground-motion parameters—which may be PGV, peak ground acceleration (PGA) or response spectral ordinates—will be dictated by the choice of parameters used to characterise the structural fragility. As noted in “Section 4.7”, when dealing with a vulnerable exposed building stock that may also be sensitive to the duration of the ground shaking—as would be the case, for example, with poorly reinforced masonry structures (e.g., Bommer et al. 2004)—then it may also be desirable to predict parameters that characterise the duration or the effective number of cycles of motion.

### Seismic hazard assessment for induced earthquakes

As explained in “Section 4.2”, risk-informed decision making requires estimates of the potential consequences of induced earthquakes coupled with their expected frequency or probability of occurrence. This leads to the requirement for probabilistic seismic hazard analysis (PSHA), which has evolved considerably since its introduction almost five decades ago (McGuire 2008). However, it has been demonstrated that the use of classical PSHA in which seismic source contributions are directly integrated leads to overestimation of risk for a spatially distributed exposure (Crowley and Bommer 2006). This leads to the necessity to use Monte Carlo approaches to PSHA, which are well established (Musson 2000; Assatourians and Atkinson 2013) even though they have not been very widely deployed for assessing the hazard from natural seismicity, possibly due to being computationally more intensive for the calculation of hazard at a single site. The Monte Carlo approach to calculating probabilistic seismic hazard actually brings several advantages, including very straightforward implementation of probabilistically defined site amplification functions (Bazzurro and Cornell 2004) and the estimation of vector hazard in which combinations of ground-motion parameters are considered (Bazzurro and Cornell 2002).

Seismic hazard models for induced seismicity will be fundamentally different from those developed for natural seismicity in the sense that the seismicity in the former case is genuinely non-stationary, so the hazard will vary with time. Time-dependent PSHA models have been developed, but these are usually based on short-term probabilities of events considering the current position in the seismic cycle (e.g., Akinci et al. 2009; Petersen et al. 2007) or as a result of Coulomb stress transfer following large earthquakes (e.g., Parsons et al. 2000). There is little possibility of developing a generalized approach to seismic hazard assessment for induced earthquakes, since the models for earthquake occurrence will vary appreciably from one anthropogenic activity to another. To date, hazard models have been proposed for geothermal projects (Convertito et al. 2012; Goertz-Allmann and Wiemer 2013; Mena et al. 2013; Hakimhashemi et al. 2014) and for a conventional gas field (Bourne et al. 2014, 2015).

Another important distinction of seismic hazard assessment for induced seismicity from conventional PSHA is the lower magnitude limit considered, *M*_min_. In standard PSHA, which is usually conducted to determine seismic design loads, the hazard integrations exclude contributions from earthquakes considered too small to generate sufficiently energetic motions to pose a threat to new constructions; values used as the lower magnitude limit are generally on the order of 4.5 to 5. For the case of induced seismicity affecting an existing building stock—which may have been constructed with no consideration at all for earthquake loading, and moreover may be in a poor state due to age and lack of maintenance—it would be indefensible to exclude such earthquakes. Indeed, it is likely that many if not most of the induced events causing concern may be appreciably smaller than the *M*_min_ values often applied in standard PSHA practice. An obvious consequence of this, however, is that direct comparison of estimated hazard levels from induced and natural seismicity, if calculated with different lower-bound thresholds on magnitude, are likely to be misleading.

### Ground motion prediction equations

An indispensable element of any seismic hazard or risk model is an equation predicting values of a particular ground-motion parameter as a function of magnitude, style-of-faulting, distance, site conditions and other variables that may characterise the earthquake source, the travel path followed by the seismic radiation and the near-surface conditions at the site. Hundreds of such GMPEs are now available (e.g., Douglas 2014), which would suggest that it might be possible to select suitable models from this list for application to induced seismicity. However, there are several obstacles to this approach, which will generally lead to the necessity to derive new GMPEs specifically applicable to each case of induced seismicity. The first issue is that GMPEs have generally been derived for the purpose of deriving input to engineering design, with the focus logically placed on larger earthquakes: The lower bounds of magnitude covered by empirical equations is often in the range of 4–5. As noted above, in the assessment of hazard and risk from induced seismicity, much smaller magnitude earthquakes are likely to be of interest. Using European strong-motion data, Bommer et al. (2007) showed that extrapolation of empirical GMPEs to smaller magnitude leads to overestimation of the ground-motion amplitudes. Subsequent studies of western North American data confirming the same effect also identified clear regional variations in the motions from smaller magnitude earthquakes that do not persist at larger magnitudes (Atkinson and Morrison 2009; Chiou et al. 2010). Whereas several studies have suggested that regional variations in ground motions among regions of shallow crustal seismicity may not be very large (*e.g*., Stafford et al. 2008), additional epistemic uncertainty may need to be considered when importing GMPEs from other regions for application to induced seismicity.

As well as being calibrated to smaller magnitudes than conventional GMPEs, models applied to induced seismicity need to be calibrated to the very shallow focal depths common for induced earthquakes. Some GMPEs have been derived covering the magnitude, depth and distance combinations relevant to induced seismic hazard analysis, using recordings from both tectonic (Atkinson 2014) and induced (Sharma et al. 2013; Douglas et al. 2013) earthquakes. A particular issue is what might be an appropriate distance metric to be used, given that induced earthquakes tend to occur at shallower depths than most tectonic seismicity. This may lead one to conclude that the use of distances measured horizontally at the Earth’s surface, such as the Joyner-Boore distance, *R*_JB_, or epicentral distance, *R*_epi_, are inappropriate since they are unlikely to capture the depth effect. However, if it is the case that shallower earthquakes are associated with lower stress drop drops (e.g., Allen 2012), then inclined distances, such as rupture distance, *R*_rup_, or hypocentral distance, *R*_hyp_, for these very shallow events may actually overestimate ground motions. Analysis of intensities from induced earthquakes in the US indicates that the two effects of shorter travel paths to the surface and reduced stress drops tend to cancel each other out in the epicentral region (Hough 2014). Since induced seismicity tends to occur in a rather narrow depth range for any given application, it may be suitable to use horizontal distance metrics provided the median motions are appropriately adjusted at short distances.

In order to allow correct sampling of the ground-motion variability (sigma) associated with the GMPE in the Monte Carlo simulations, it is a requirement that the GMPE be derived in a manner that quantifies the between-event and within-event components of sigma (Al Atik et al. 2010).

Another consideration is that if, as suggested in the next sub-section, fragility functions are to be defined as functions of both ground-motion amplitude and duration, then GMPEs are also required for the latter. The joint (or vector) prediction of amplitude and duration needs to account for any negative correlation of the residuals—such that motions with exceptionally high accelerations would tend to be associated with unusually short durations—as has been proposed, for example, by Bradley (2011).

### Fragility functions

**Fig. 4:**
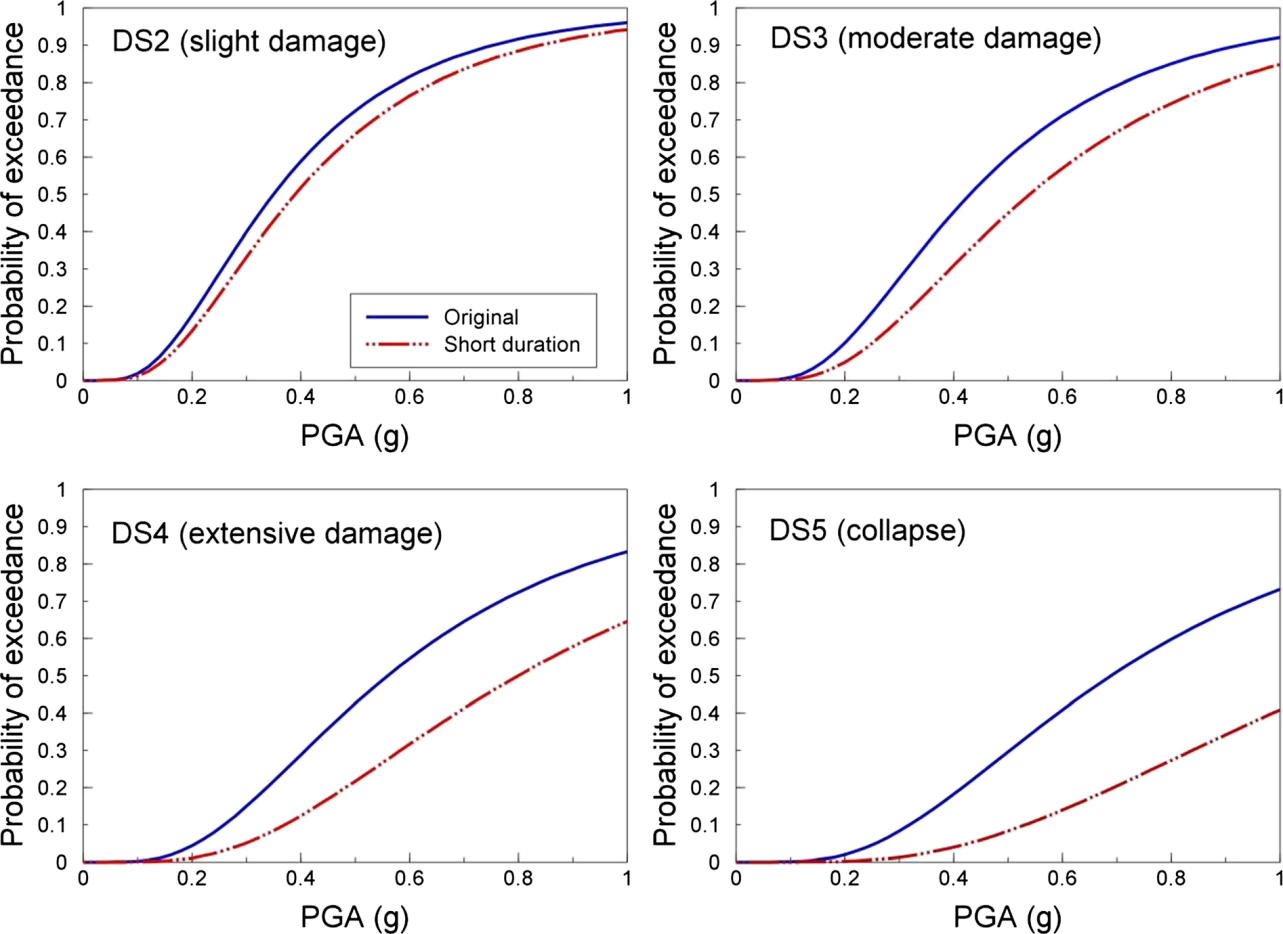
Representation of the potential influence of short ground-motion duration on fragility functions for different thresholds to damage (damage states DS2 to DS5)

Fragility functions define the probability of exceeding limit states to damage, conditional on a level of ground motion intensity (see Fig. 4). There are a number of different approaches that can be applied in the derivation of fragility functions, depending on the availability of data, time and resources. Empirical methods make use of existing post-earthquake damage data to correlate the observed levels of damage (to the elements at risk) with the estimated ground shaking intensity to which they were subjected (e.g., Whitman et al. 1973; Colombi et al. 2008). Although they provide an insight into the actual behaviour of structures and infrastructures to ground shaking, there are a number of drawbacks to using empirical methods for deriving fragility functions, including the fact that post-earthquake damage data are often biased as it is frequently collected where the most damage is observed, undamaged structures are often not included in the data and recordings of ground motion for the damaged region are often not available. Studies suggest that, unless there is a dense network of strong-motion instruments, the inherent variability in ground motions renders correlations of observed damage with shaking intensity very uncertain (Crowley et al. 2008). Analytical methods use numerical simulations of the built environment to estimate the response of the structures/infrastructures to increasing levels of seismic excitation (e.g., Singhal and Kiremidjian 1996; Silva et al. 2013). This response then needs to be correlated with damage, which is one of the main challenges of analytical methods, as this translation often requires engineering judgement or experimental data on many structural and non-structural components. Experimental tests of full-scale structures can also provide extremely useful input for the derivation of fragility functions (e.g., Bothara et al. 2010), but it would be too expensive and time-consuming to use laboratory tests to model all of the uncertainties and ground-motion intensities necessary to constrain a fragility function.

Many fragility functions have been developed over the years (e.g., Calvi et al. 2006), but the majority of available functions are not appropriate for use in an induced seismicity-based risk assessment. Even if fragility functions have been published for similar building typologies to those found within the region of induced seismicity (in terms of material, structural system, height, code design), they are likely to have been derived considering ground motions with different characteristics to those expected from induced seismicity events. In particular, the magnitude of the events used in the derivation of fragility functions can have a significant effect on the damage estimation, due to the influence of magnitude on both spectral shape and duration; given that the vast majority of fragility functions have not been derived considering induced earthquakes, they are likely to have used ground motions with much higher magnitudes and longer durations. As noted earlier, Bommer et al. (2004) have demonstrated the impact of strong ground-motion duration on the response of unreinforced masonry buildings, whilst Chandramohan et al. (2013) have recently shown how fragility functions for steel moment frame buildings are strongly dependent on the ground-motion duration. These structures exhibit a deterioration of the stiffness and strength with increased cyclic behaviour, and thus, a record of long duration is more likely to subject the structure to sufficient inelastic deformation that could induce collapse. For the lower levels of damage, which occur under much lower levels of inelastic deformation (and thus before there are significant reductions in stiffness and strength), the impact of duration is likely to be much less pronounced. It would thus be expected that fragility functions for different thresholds to damage (from DS2, which is a slight damage state, to DS5, which refers to collapse) would be affected by the shorter duration of induced seismicity events in the manner presented in Fig. 4. This figure is illustrative, but the fragility functions labeled ‘original’ in this figure could be derived through nonlinear dynamic analysis for a given structural model using sets of accelerograms, where each set is scaled to increasing levels of intensity (in this case PGA). At each PGA level, the percentage of accelerograms that cause a given damage state (from 2 to 5) to be exceeded would be calculated and plotted against the value of PGA. By repeating these analyses with other sets of records with similar spectral properties, but much shorter durations (e.g., Hancock and Bommer 2007), we would expect to obtain the ‘short duration’ labeled fragility functions in Fig. 4. A vector-based approach for deriving fragility functions (e.g., Gehl et al. 2013), wherein the probability of exceeding the damage state is conditioned on both a level of ground shaking intensity (e.g., PGA, PGV, spectral acceleration at the fundamental period) as well as the duration of strong ground shaking (e.g., 5–75 % significant duration) thus merits further research for application in induced seismicity-based risk assessments.

Given the need to consider different characteristics of the ground shaking when deriving fragility functions for induced seismicity applications, and the possibility that the buildings may have characteristics that have not been considered in previous fragility studies, the most appropriate path to follow for this component of the risk model would be to derive application-specific fragility functions for the buildings located within the vicinity of the project. These fragility functions should be derived using an analytical methodology, in order to allow the characteristics of the buildings to be explicitly included in the numerical models, and for ground motions that are compatible with the hazard in the region (in terms of spectral content, duration, correlation of intensity measures, record-to-record variability; see, for example, Bradley 2010) to be employed in nonlinear dynamic analysis. Should additional time and resources be available, experimental tests of the materials, structural components or even full-scale buildings, could be carried out to calibrate further the numerical models. Finally, once a first set of fragility functions has been derived, consistency checks using good quality post-earthquake damage data for similar building types can be carried out, acknowledging the differences that would be expected if the characteristics of the buildings and earthquakes differ. With such an approach, the strengths of each previously described methodology for deriving fragility functions can be exploited.

The derivation of application-specific fragility functions allows a greater focus to be placed on the damage states that are more relevant for the selected risk metrics (see “Section 4.2”). For example, should the most appropriate risk metric be location risk, more emphasis should be placed on the estimation of significant damage and collapse of the buildings within which people spend the majority of their time, given that these are the damage states that pose the highest threat to life.

Another issue that is worth considering when developing fragility functions for induced seismicity applications is the effect of cumulative events on the response of the structures. The exposed assets may be subjected to many induced events of low to moderate magnitude causing minor levels of damage to the buildings that cannot always be repaired ahead of the next episode of shaking. This pre-existing damage will affect the strength and stiffness of the buildings, and have an influence on the response of the structures. Again, this issue might only be of importance for some risk metrics, as the effect of moderate pre-existing damage on the buildings is likely to influence the lower damage states more than collapse (e.g., Abad et al. 2013).

One more issue to be kept in mind is the influence of the lower end of the fragility curves. The general use of a log-normal distribution (which is a convenient rather than necessary assumption) means that, for low levels of acceleration, there will be non-zero probabilities of reaching each damage state, including the more serious DS4 and DS5 levels. If the risk calculations are performed for a large exposure of tens of thousands of buildings, with many simulated earthquake catalogues, it is possible that these tails on the fragility curves will lead to non-zero estimates of casualties even for very low shaking levels. This may justify modification of the lower end of the fragility curves, particularly for the higher damage states, to avoid this unintended consequence.

### Calculation of risk

A probabilistic seismic risk assessment is required to estimate the probability that the loss due to damage from ground shaking (or other seismic hazards) will equal or exceed specified values (at a site, at several sites, or in an area) during a specified exposure time. Given that the assets exposed to induced seismicity are likely to be spread over an extended region, it is necessary to simultaneously model the levels of ground shaking at each site within the exposure model for each event that could occur within the selected exposure time. As mentioned previously, Monte Carlo simulation is frequently used for this purpose given its simplicity: Events are randomly sampled for each seismic source from their magnitude–frequency distribution, and the ground motions at all sites of the model are estimated from ground-motion prediction equations, with a random sample of the inter-event (between earthquake) variability for the event and a random sample of the intra-event (within earthquake) variability for each site (e.g., Crowley and Bommer 2006). An important consideration to make when modelling the ground shaking at each site is the cross-correlation between the residuals of the ground-motion prediction equations for the different intensity measures that might be needed for the fragility functions (e.g., PGV and duration), as well as the spatial correlation of the residuals of these intensity measures at two different sites, which is a function of their separation distance (e.g., Crowley et al. 2008; Weatherill et al. 2013).

In order to simplify the computational burden that accompanies the estimation of spatially cross-correlated residuals at a large number of locations for tens of thousands of events, it might be necessary to aggregate the assets within grid cells and assume full spatial correlation of the ground motion residuals within the grid cell and no spatial correlation between grid cells. This simplification is less straightforward when vector-based intensity measures are used for the fragility functions, as the spatial correlation of each intensity measure will correlate differently with distance, and so the optimal grid size might vary for each intensity measure. Other factors that might be considered when defining the size of the grid include the resolution of the available data on site amplification and the density of the buildings. A variable grid size could be employed for further computational efficiency, with smaller grid cells where there is a higher density of buildings and site amplification factors. Sensitivity studies, with and without correlation of the intensity measures and with variable grid cells, can be undertaken to investigate the impact of the exposure resolution and correlation on the resulting risk results (e.g., Bal et al. 2010; Bazzurro and Park 2007).

One of the main advantages of using Monte Carlo simulation to obtain damage and loss estimates as outlined above is that it allows the loss, damage and hazard to be easily disaggregated, and to thus identify the events (e.g., in terms of magnitude and location or shaking levels), the assets, and the damage states that are contributing most to the loss. Disaggregation of the risk is the best practice to obtain single scenario events that can then be investigated in further detail, for the purposes of risk communication or emergency planning, as discussed previously. The process by which damage and loss should be estimated for a single event is similar to that described above, the only difference being that the given event will need be repeated many times in order to fully sample the inter- and intra-event variability in the ground-motion prediction equation, as well as the uncertainty in the fragility functions and consequence models. The mean and standard deviation of the damage and loss statistics can then be presented for the selected scenario event.

### Updating hazard and risk assessments

**Fig. 5:**
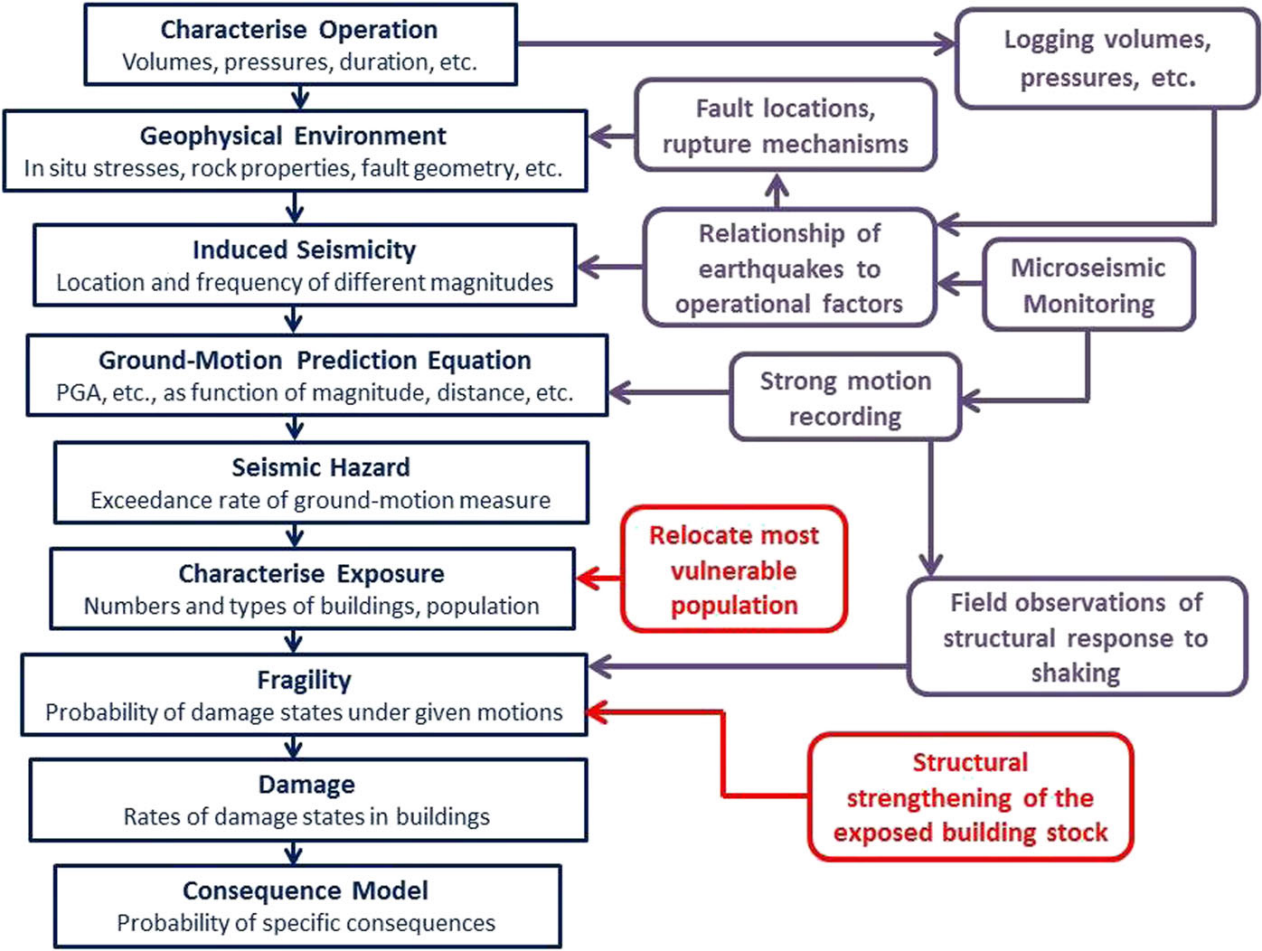
The sequence of steps involved in estimation of induced seismic risk and opportunities for updates of the hazard and risk models. Those elements in *purple* represent potential reductions in epistemic uncertainty through new data collection whereas those in *red* are modifications to the risk through mitigation measures (control of the hazard through ‘traffic light’ systems is not included)

Seismic hazard assessments conducted to estimate the ground shaking from tectonic earthquakes can generally only be improved after the accumulation of new data, which implies long waiting times. Even for critical facilities such as nuclear power plants, updates of seismic hazard estimates are generally only required every decade. In the case of induced seismicity, as soon as operations commence, provided that adequate monitoring networks are in place, the frequency of induced events will often allow rapid, and repeated, updates of the hazard and risk models. This is very valuable, since the epistemic uncertainty in such models developed *a priori* is likely to be high, and all new data collection allows for greater constraint and reduced overall uncertainty. Some of the options for reducing the uncertainty are illustrated in Fig. 5.

Microseismic monitoring enables high-precision location of induced earthquakes and determination of focal mechanisms, which can provide insights regarding the faults that may be present and the stress field (e.g., Deichmann and Ernst 2009; Dinske and Shapiro 2013; Kwatiek et al. 2014). If the parameters that define the operational activity—such as fluid pressures and pumped volumes—are also monitored, then the model relating the seismicity to the activity can be refined.

If strong-motion accelerographs are also installed as part of the monitoring programme, then the GMPE can also be updated; this is a particularly important element of the updating since uncertainty in the median ground-motion predictions is likely to be a significant source of uncertainty. For the Berlín geothermal project in El Salvador, for example, Bommer et al. (2006) originally adopted a GMPE for PGV calibrated using recordings from earthquake swarms in the region; once a sufficient body of data from the induced earthquake became available, the GMPE was updated to fit the new local recordings.

With a very dense array of strong-motion instruments (such that it is reasonable to assume uniform amplitudes of motion in conjoining areas surrounding the instruments), field observations of structural response following episodes of felt shaking may also allow calibration or verification of the fragility curves, at least for the lower damage states. However, this may be complicated by the fact that after experiencing minor or moderate damage, the fragility of the buildings may also be modified. In practice, there is far greater scope for reduction in the uncertainty associated with the fragility through extensive structural analyses, supplemented by testing of building materials and, where possible, structural specimens.

Clearly, the opportunity to make use of data gathered from small induced earthquakes to update the hazard and risk models, presupposes that the operations are allowed to proceed following minor shaking episodes. If the operations are suspended—as happened to the first shale gas project in the UK, where hydraulic fracturing led to a magnitude 2.3 earthquake in 2011—then the opportunity is clearly lost.

In addition to the options for updating the hazard and risk estimates as new data become available, revised estimates of the risk can be used to quantify the impact of different mitigation measures. For example, if it were decided that it were both desirable and feasible to relocate, to new dwellings or a new location, people living in the most vulnerable buildings—for which effective seismic retrofit proves very difficult and expensive—then the impact on the risk could be very easily estimated with new runs of the model. The most powerful and useful application of iterative loss modelling, however, would be to explore the impact of different schemes of structural strengthening in order to optimise a programme of interventions that would lead to the desired reduction of risk. This application of iterative loss modelling is discussed further in the next section.

## Engineering risk mitigation measures

**Fig. 6:**
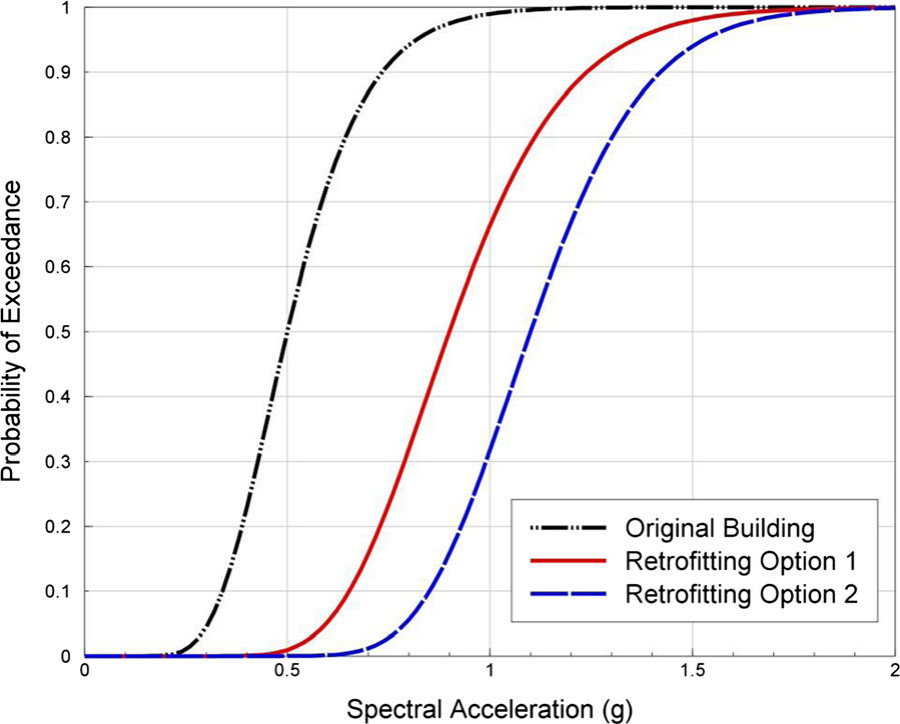
Fragility functions for a building retrofitted to two different levels of seismic capacity

As stated previously, it is our view that the most effective manner in which the risk posed by potential induced ground shaking may be mitigated is through seismic upgrading interventions aimed at increasing the earthquake resistance of existing structures. The impact of such structural interventions can be readily identified in fragility functions (Fig. 6) and, subsequently, duly considered in the type of cost–benefit risk analyses discussed below.

Before addressing the details of structural strengthening for seismic resistance, it is worthwhile recalling that the focus here is exclusively on structural damage that could pose a threat to life and limb. As has been noted previously, upgrading buildings to prevent minor non-structural damage in the form of plaster cracks and the like would probably be prohibitively expensive and therefore not cost-effective. However, since the focus is very much on protecting occupants and passers-by from injury, attention should obviously also be given to important non-structural elements, such as chimneys, which could pose a serious falling hazard.

### Performance targets for existing structures

Our understanding and awareness of how damaging earthquake actions can be on the built environment is today much higher than it was a few decades ago, whilst the tolerance of modern society with regards to the consequences of such damage is considerably lower. As such, present regulations for seismic design of new structures around the world inevitably impose significantly stricter performance requirements than those that were considered when designing and constructing structures in the past. This effectively means that it is often unfeasible for older buildings to be made to comply, through structural upgrading, with such modern seismic performance requirements for new structures; attempting to do so would be counter-productive, since the ensuing unsustainable costs would unavoidably lead to a ‘do-nothing’ outcome (Fardis 1998).

Therefore, and as discussed in Grant et al. (2007), it is widely acknowledged in earthquake engineering practice around the world that for retrofitted buildings lower levels of seismic capacity than would be required in new buildings can be accepted. The ATC 3–06 guidelines (ATC 1978) pioneered the definition of such seismic performance targets specifically for existing buildings, based on the concept of tolerable risk levels that are a result of the need to strike a balance between the reduction of vulnerability and the cost of rehabilitation. An earthquake capacity ratio, *r*_*c*_, defined as the ratio between the strength of the existing building and that of a new code-compliant building, was allowed to take values below unity, effectively meaning that existing buildings can have a seismic capacity that is less than that required for new design (i.e., *r*_*c*_ < 1.0). For buildings that are essential for post-earthquake recovery, ATC 3-06 specified a minimum capacity ratio (*r*_*c*,min_) of 0.5 (i.e., half the capacity required for new design) whilst, for buildings that are of lesser importance, the threshold is given as a function of the potential number of occupants but can be as low as 0.25.

In New Zealand, the guidelines *Assessment and Improvement of the Structural Performance of Buildings in Earthquakes* (NZSEE 2006) follow a similar rationale, defining a tolerable seismic risk for existing buildings, whereby a building with a seismic capacity below 33 % of the new building design level requires seismic retrofitting, whereas between 33 and 67 %, retrofitting is simply recommended. Hence, a higher level of risk is accepted for existing buildings, due to the unacceptable cost of bringing them to the same standard as required for new buildings. Existing buildings are thus expected to reach the limit states defined for new buildings under lower levels of ground motion, which in turn will have lower return periods. Noteworthy is the fact that buildings retrofitted to the 33 % level were seen to perform relatively well, in terms of life safety, during the 2011 Christchurch earthquake in New Zealand (Ingham and Griffith 2011).

The 2009 revision to the NEHRP Provisions (NEHRP 2009) introduced a new conceptual approach to the definition of the input seismic action for design and assessment (as described in Luco et al. 2007). An acceptable probability of collapse is predefined, and then the levels of hazard that would lead to this uniform probability of collapse are back-calculated (this effectively leads to a variation of the return period considered for the ground motions across the region of interest). An earlier work by Bommer et al. (2005), however, had gone one step further, by proposing the consideration of local/regional risk evaluation for the assessment of existing structures, whereby decision makers weigh the cost of different retrofitting schemes (with varying details) against the subsequent annual average losses that would be expected for each.

Crowley et al. (2012) explored further, through a case-study application, the aforementioned conceptual approach initially put forward by Bommer et al. (2005), confirming the feasibility of undertaking this type of cost–benefit analysis at a regional scale in order to define the optimum seismic resistance capacity level that buildings should be made to possess. This methodology is particularly appealing for the case of induced ground shaking, since it does away with the need to couple structural performance targets with specific seismic action levels associated to return periods set somewhat subjectively by code drafting committees (Bommer and Pinho 2006).

Clearly, risk metrics other than direct costs may also be considered in the undertaking of cost–benefit analyses aimed at defining the desired seismic performance level of the built environment in an area exposed to potential induced ground shaking. These could include location or group risk, already discussed in “Section 4.2”, as well as indirect losses, nuisance to the population and reputational risk, with the recognition that the inclusion of the latter in the quantitative framework described herein would be more challenging.

### Matching performance targets with structural interventions

There is extensive literature on repair and strengthening methods for structures subjected to earthquakes, an area in which researchers and practitioners have been particularly active for some decades. For instance, in 1980, a first workshop on seismic retrofitting of existing structures was organised under the framework of the US/Japan Co-operative Earthquake Engineering Research Program (Hanson 1980), whilst in 1986 the Japanese Ministry of Construction summarised all research in this field in a single comprehensive volume (PWRI 1986). Several state-of-the-art reviews on repair and strengthening have been published since then, pioneered amongst others by the likes of Jirsa and Kreger (1989), Bertero (1992), Sugano (1996), and many special issues in international journals have also been released, with one of the first being the *Earthquake Spectra* issue on Repair and Rehabilitation Research for Seismic Resistance of Structures (EERI 1996).

**Fig. 7:**
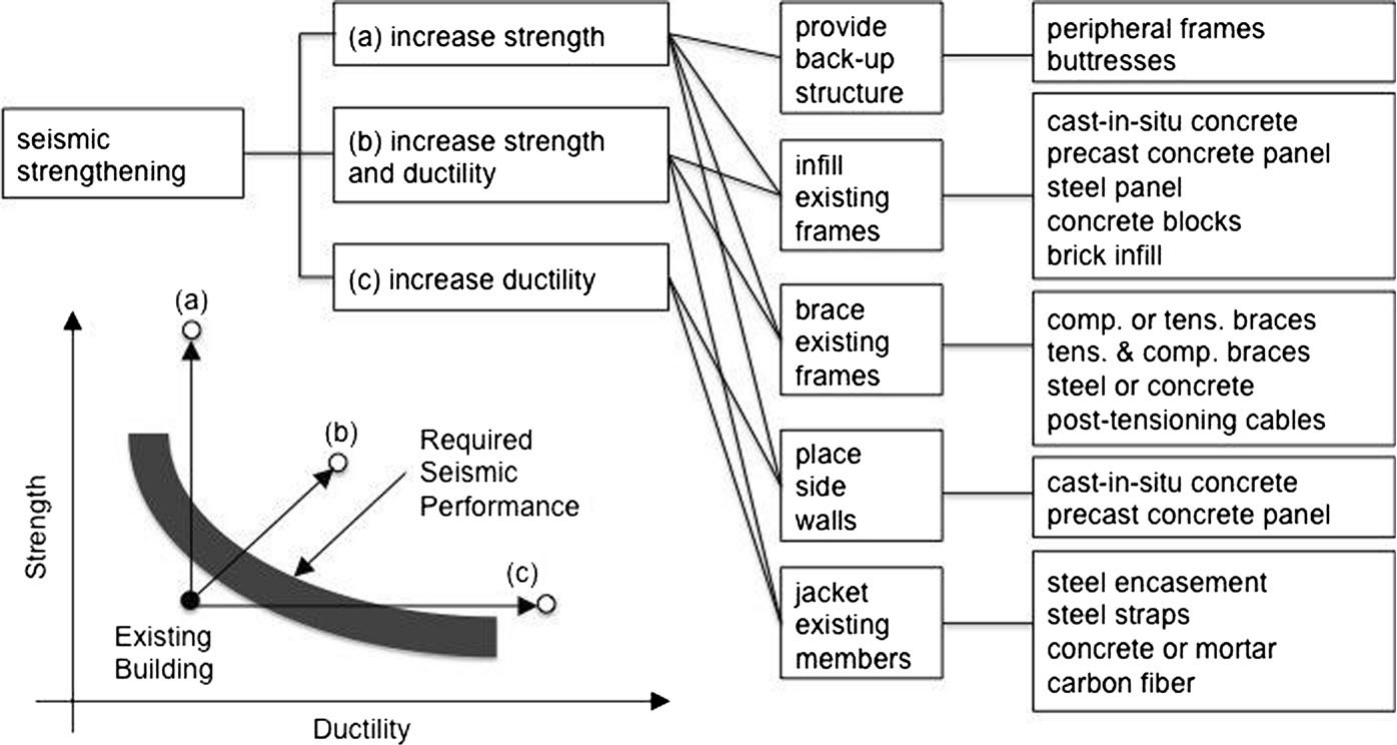
Typical structural intervention techniques (redrawn from Sugano 1996)

In Fig. 7, a summary of typical intervention techniques is presented, together with a schematic representation of the redesign scenarios where these are most likely to be employed. Such methods may be subdivided into global and element intervention types. Examples of the former include peripheral frames and buttresses whilst the latter can take the form of member jacketing or injection of epoxy resin.

The conceptual strength versus ductility relationship shown in Fig. 7 renders also evident that targeting the upgrade of a particular structural response parameter, such as ductility (i.e., the capability for a structure to deform inelastically without loss of strength), may call for the employment of an intervention approach that is markedly distinct from that used when upgrading a different structural response parameter, such as strength (i.e., amount of seismic force that a structure may withstand with minor or no damage).

By the same token, different seismic performance targets, as defined through the type of cost–benefit analyses described above, will require the application of varying structural intervention schemes. Indeed, and as an example, whilst guaranteeing that an existing building will not experience partial collapses under seismic action (e.g., aimed at reducing location risk) may call for wrapping of its structural elements using carbon-fibres, the avoidance of any damage to structural and non-structural elements (e.g., aimed at reducing business interruption) may instead require the addition of lateral buttresses or base isolation.

As noted in “Section 2.3”, it is recalled, however, that the selection of a given structural intervention is not necessarily driven solely by the need to meet a certain performance target. Indeed, other factors such as the availability of workmanship (e.g., advanced retrofitting techniques may require know-how not locally available), disruption to occupants and the use of the building (e.g., introduction of base isolation will require full evacuation from the structure for a prolonged period of time), architectural impact (e.g., addition of external steel braces may contrast excessively with the aesthetics of local construction), community perception (e.g., ‘light’ internal structural interventions may leave some homeowners feeling not safeguarded enough, whilst highly visible and invasive external interventions may give rise to fears of loss of market value for the property), etc., will also need to be duly accounted for when planning and deploying a seismic upgrading campaign.

### Prioritisation and scheduling

When a relatively large number of buildings or houses are deemed to require seismic upgrading, the need for prioritisation and scheduling (e.g., Grant et al. 2007) is unavoidable. Indeed, even if an unlimited budget were to be available, it would hardly be feasible to (1) secure the human resources (engineers, architects, builders, etc.) required to undertake the seismic retrofitting of hundreds or thousands of buildings, and (2) find alternative accommodation/workspace for the thousands of individuals that would have to be temporarily relocated.

The first step in such a decision-making process is clearly that of ranking the buildings in decreasing order of the computed seismic risk, which, as discussed above, will require the selection of a given risk metric (or appropriate combination of different risk metrics). Once such prioritisation ranking is established, the second step of the process will be that of defining the financial resources available for retrofitting and the levels of risk that may be considered tolerable, so that the scheduling of the strengthening campaign may be set.

Clearly, the above decisions will call for extensive discussion and consultation amongst different stakeholders, including regulators, local authorities and local population. Whilst this is bound to be a difficult decision, since different choices may lead to different relative prioritisation/scheduling for retrofitting, it is critical that risk mitigation activities are not unduly delayed by such consultation process, and that thus executive and pragmatic decisions are taken, so that the first seismic upgrading activities may start without delay (with allowance for continued revisiting and progressive adjustment of the prioritisation and scheduling programme).

By the same token, the process of identifying and designing the most appropriate seismic upgrading schemes, considering both the structural performance objectives as well as all the other non-technical factors described in “Section 5.2”, should not lend itself to excessively lengthy discussions and iterations that delay the start of the seismic upgrading of those buildings ranked higher in the prioritisation list. This might imply the need to adopt a pragmatic approach, whereby even preliminary non-engineered structural retrofitting measures (e.g., floor ties, chimney restraints, etc.) start being introduced as soon as possible by local contractors, whilst the iterative–incremental process of refining the design of definitive strengthening measures and securing construction materials and the additional specialised workforce potentially required continues.

## Conclusions and recommendations

This paper has discussed options for mitigating the risk associated with induced seismicity. The paper is not intended to be prescriptive but rather to initiate discussion of the available options and to present new perspectives, specifically recognising the opportunities for risk mitigation through modification of all of the elements of risk—including exposure and vulnerability—rather than only attempting to control the hazard, which has been the main focus of most work in this area to date. This is not to say that attempts to regulate the causative operation to limit the number or size of induced earthquakes has no place in the management of the consequent risk, but we believe that it may not be optimal approach in many cases, not least because of the inherent uncertainty and the unproven reliability of traffic light systems in practice.

**Fig. 8:**
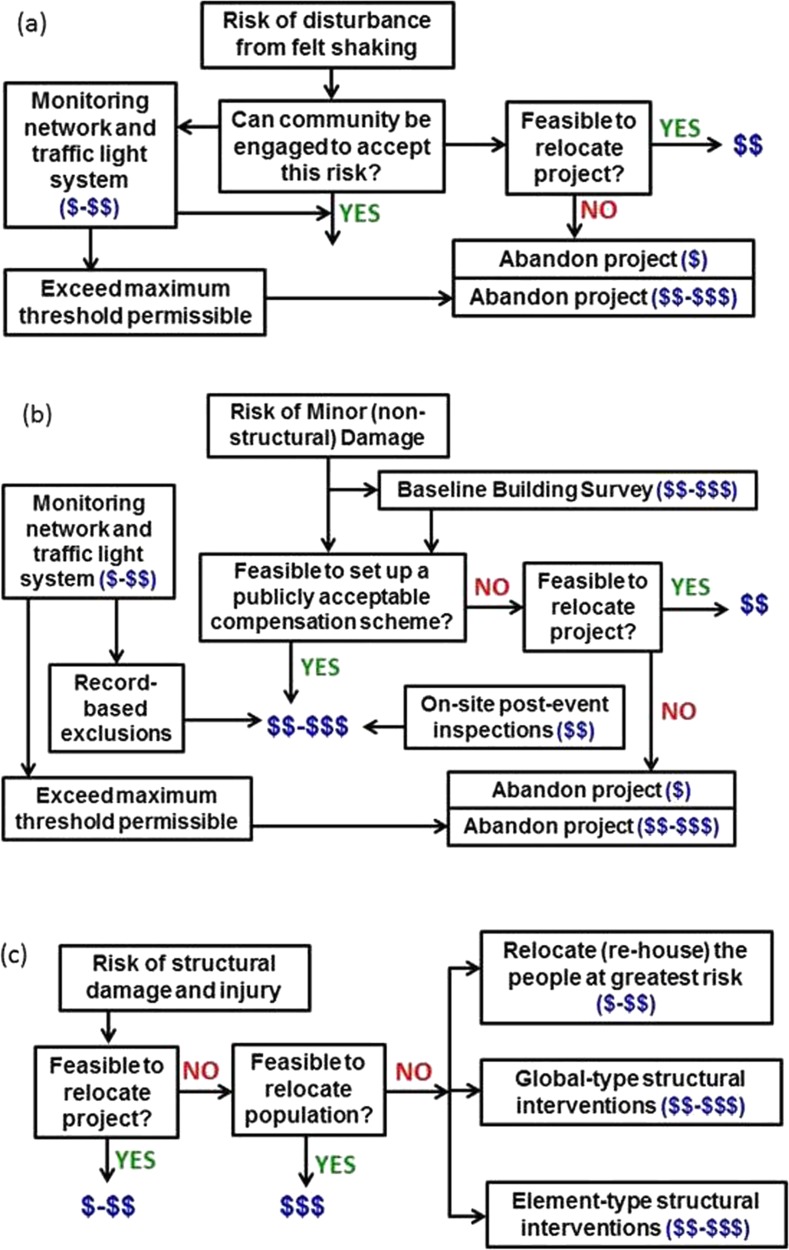
Flowcharts indicating suggested options for managing risks of **a** felt shaking causing annoyance, **b** non-structural damage incurring repair costs, and **c** structural damage that could threaten life and limb. In each case, range of possible costs associated with each alternative are indicated ($: low; $$: medium: $$$: high)

Figure 8 illustrates possible responses to different levels of risk due to induced earthquakes. In the case of genuinely small earthquakes, the risk will be primarily that of felt shaking episodes (Fig. 8a). The consequences will be annoyance to the affected population, potentially heightened by the fear that the moderate shaking events will be precursors to stronger tremors. Numerical quantification of this risk is extremely difficult because of the numerous subjective factors contributing to what is an acceptable intensity and frequency of being shaken (e.g., Douglas and Aochi 2014). The potentially most beneficial approach in such a case is to engage with the affected population in order to attain support for (or at least tolerance of) the project, based on benefits to the local community or to the environment. In such cases, the implementation of an instrumentally based traffic light scheme may be very useful in providing additional assurance to the community. If the community’s support cannot be garnered, then it may be necessary to relocate the project, if this is feasible. However, for a geothermal project, for example, the location will be controlled partly by where there is access to crustal heat, and if the project is remote from conurbations then it is unlikely that there will be the economic benefit from also providing district heating. Deciding to abandon the project on the basis of an *a priori* risk assessment will incur costs due to lost investment, but these may be modest in comparison to those resulting from suspension of the project in the event of the highest thresholds on the traffic light being exceeded. In the latter case, there will also be a significant loss of trust and public confidence. In this regard, it is possible that cases such as Basel have already jeopardised the long-term feasibility of enhanced geothermal systems, since any population faced with such a project will look at the experience in the Swiss city. Although cogent arguments have been put forward regarding the need to accept the seismic risk from such projects (Giardini 2009), it is not clear that the public is yet persuaded that the benefits of this renewable energy source outweigh the threat posed by induced earthquakes.

If the risk assessment indicates that as a result of stronger shaking minor—but nonetheless troublesome—damage may result in the form of plaster cracks and other non-structural consequences (Fig. 8b), different responses are required. In such a case, once again public engagement is essential but acceptance is unlikely without a clear agreement to provide adequate compensation. In other regards—implementing a traffic light and considering relocation—the issues are similar to those faced in the previous case. For the compensation scheme, the cumulative costs could be very high. There are options that can help to reduce the pay-outs, including a survey to establish the baseline condition of the exposed buildings (if house owners grant access) and then carrying out on-site inspections of reported damage. Although these measures may reduce compensation payments, they will be expensive in themselves, with the potential added disadvantage of undermining the relationship of trust with the affected community. Another potential device to avoid unnecessary payments would be to use the instrumental traffic light system to establish exclusionary criteria against new claims (based on excessively low amplitudes of shaking). Here again, however, the benefit of the saving could be outweighed by the negative impact on the relationship with the affected community, unless the exclusionary thresholds are set very low.

In the worst scenario, the strength of the expected shaking combined with the poor seismic resistance of the exposed building stock can result in the possibility of structural damage with the consequent risk of injury or death (Fig. 8c). In these cases, we believe that traffic light systems are not yet sufficiently reliable to be depended upon, given the unacceptable consequences of their failure; this does not, of course, preclude the need for instrumentation, both for micro-seismic monitoring and strong-motion recording. Since the costs of risk reduction are inevitably high, relocation of the project must be seriously considered, and here, there is likely to be an element of natural selection. For operations such as wastewater disposal and carbon storage, which will be associated with small profit margins or even net costs, the logical choice would be to seek alternative locations. For hydrocarbon extraction, relocation is impossible, but, at the same time, the profit margins are likely to make engineering risk mitigation measures possible; if not, then abandonment of the project may be the only solution. The safest solution is to relocate the population, but this is likely to be very expensive and controversial—as shown by the renewed attempts to relocate villages for lignite mining in Germany—if feasible at all. If neither the project nor the population can be moved, then a series of targeted and intelligently prioritised measures can be taken to reduce the risk to acceptable levels. For those people located in high hazard areas in extremely vulnerable buildings, the ideal solution is likely to be rehousing. For others, the various engineering interventions outlined in “Section 5” can be implemented.

There are two key points that we have attempted to emphasise in this paper. The first is that in order to develop an effective response plan for dealing with potential induced seismicity, the starting point should be to properly quantify the risk, understood as the convolution of hazard, exposure, fragility and consequence models. The tools and procedures that have been developed for the estimation of seismic risk due to tectonic earthquakes may be adopted for this purpose, but these require several modifications in order to be applicable to induced seismicity. Although uncertainties in such models will generally be high, an advantage is presented in the case of induced rather than natural seismicity, namely that new data should become available as operations proceed, thus allowing frequent updating of the risk model with better constraints.

Our second point is that once a risk model is established, it provides a rational basis for decision making regarding mitigation measures. If the estimated risk—including due account for the influence of uncertainties—is viewed to be unacceptably high, then mitigation measures are needed. For the elements of the built environment that will be exposed to the potential shaking hazard, the greatest assurance of reduced risk can be achieved through structural strengthening to reduce seismic vulnerability. Using a well-calibrated risk model, iterative calculations can be used to ascertain the modifications to the fragility functions required to attain an acceptable risk level. A retrofit scheme for each building class that achieves the identified fragility improvement can then be designed. Even in the face of appreciable uncertainty in the baseline risk model, the relative benefit of different mitigation approaches can be assessed with some confidence.

Economically acceptable adjustments to the operations causing the earthquakes that can effectively limit the induced seismicity and hence the seismic hazard may be an attractive approach to the risk management, and we fully support ongoing work to develop and refine such procedures. Our view, however, is that currently the confidence with which the levels of induced seismicity can be controlled—particularly for operations that do not involve high-pressure fluid injections—is sufficiently low that, even if ‘traffic light’ approaches are used, it is advisable to still employ strengthening measures in the most vulnerable or exposed buildings.
